# A phase II trial of gefitinib as first-line therapy for advanced non-small cell lung cancer with *epidermal growth factor receptor* mutations

**DOI:** 10.1038/sj.bjc.6603393

**Published:** 2006-10-17

**Authors:** H Asahina, K Yamazaki, I Kinoshita, N Sukoh, M Harada, H Yokouchi, T Ishida, S Ogura, T Kojima, Y Okamoto, Y Fujita, H Dosaka–akita, H Isobe, M Nishimura

**Affiliations:** 1 First Department of Medicine, Hokkaido University School of Medicine, North 15, West 7, Kita-ku, Sapporo 060-8638, Japan; 2 Department of Medical Oncology, Hokkaido University School of Medicine, North 15, West 7, Kita-ku, Sapporo 060-8638, Japan; 3 Department of Respiratory Medicine, Hokkaido Cancer Center, 4-2 Kikusui, Shiroishi-ku, Sapporo 003-0804, Japan; 4 Department of Respiratory Medicine, Fukushima Medical University, 1 Hikarigaoka, Fukushima 960-1295, Japan; 5 Department of Respiratory Medicine, Sapporo City General Hospital, North 11, West 13, Chuo-ku, Sapporo 060-8604, Japan; 6 Department of Medical Oncology, KKR Sapporo Medical Center, 1-6 Hiragishi, Toyohira-ku, Sapporo 062-0931, Japan; 7 Department of Respiratory Medicine, Asahikawa City General Hospital, 1 Kinseicho, Asahikawa 070-8610, Japan; 8 Department of Respiratory Medicine, Dohoku Hospital, 7 Hanasakicho, Asahikawa 070-0901, Japan

**Keywords:** gefitinib, non-small cell lung cancer (NSCLC), epidermal growth factor receptor (EGFR), mutation, first-line therapy

## Abstract

Retrospective analysis has shown that activating mutations in exons 18–21 of the epidermal growth factor receptor (EGFR) gene are a predictor of response to gefitinib. We conducted a phase II trial to evaluate the efficacy and safety of gefitinib as first-line therapy for advanced non-small cell lung cancer (NSCLC) with *EGFR* mutations. Patients with stage IIIB or IV chemotherapy-naïve NSCLC with *EGFR* mutation were treated with 250 mg gefitinib daily. For mutational analysis, DNA was extracted from paraffin-embedded tissues and *EGFR* mutations were analysed by direct sequence of PCR products. Twenty (24%) of the 82 patients analysed had *EGFR* mutations (deletions in or near E746-A750, *n*=16; L858R, *n*=4). Sixteen patients were enrolled and treated with gefitinib. Twelve patients had objective response and response rate was 75% (95% CI, 48–93%). After a median follow-up of 12.7 months (range, 3.1–16.8 months), 10 patients demonstrated disease progression, with median progression-free survival of 8.9 months (95% CI, 6.7–11.1 months). The median overall survival time has not yet been reached. Most of the toxicities were mild. This study showed that gefitinib is very active and well tolerated as first-line therapy for advanced NSCLC with *EGFR* mutations.

Lung cancer has long been the leading cause of cancer death in North America and became the leading cause of cancer death in Japan in 1998 (Kuroishi *et al*, 1999). Both platinum-based and taxane-based chemotherapy offer modest efficacy and survival advantages over best supportive care (BSC) alone for chemotherapy-naïve patients with advanced non-small cell lung cancer (NSCLC). Nonetheless, objective response rate (RR) is at most 30–40%, and median survival time (MST) is only 8–10 months owing to frequent recurrence and metastasis (Non-Small Cell Lung Cancer Collaborative Group, 1995; Schiller *et al*, 2002). Other promising drug therapy has therefore long been awaited.

The epidermal growth factor receptor (EGFR) is a 170 kDa tyrosine kinase (TK) that dimerises and phosphorylates several tyrosine residues upon binding of several specific ligands. These phosphorylated tyrosines serve as the binding sites for several signal transducers that initiate multiple signalling pathways which lead to cell proliferation, differentiation, migration and metastasis, angiogenesis and antiapoptosis (Arteaga, 2002). Because EGFR is highly expressed in 43–89% of NSCLC (Scagliotti *et al*, 2004), EGFR-tyrosine kinase inhibitors (EGFR-TKIs) such as gefitinib and erlotinib have emerged as particularly promising target drugs for treating NSCLC.

Two phase II trials for patients previously treated with chemotherapy, the Iressa Dose Evaluation in Advanced Lung Cancer (IDEAL)-1 and -2, revealed favourable objective RRs of 10–20% and a disease control rate of 50% (Fukuoka *et al*, 2003; Kris *et al*, 2003). Subsequent randomised phase III trials were conducted; the Iressa NSCLC Trial Assessing Combination Treatment (INTACT)-1 and -2, which were randomised, placebo-controlled trials of cisplatin/gemcitabine or carboplatin/paclitaxel with or without gefitinib for chemotherapy-naïve patients. However, these did not indicate any additional benefit of gefitinib over conventional cytotoxic chemotherapy, as measured by RR and median and 1-year survival (Giaccone *et al*, 2004; Herbst *et al*, 2004). Moreover, another phase III trial, the Iressa Survival Evaluation in Lung Cancer (ISEL), showed no survival benefit of gefitinib over BSC as a salvage regimen (Thatcher *et al*, 2005). On balance therefore, the usefulness of gefitinib for advanced NSCLC remains controversial. Similar results were observed regarding another EGFR-TKI, erlotinib (Gatzemeier *et al*, 2004; Herbst *et al*, 2005), with the exception of the BR21 trial which showed survival benefit over BSC for erlotinib as salvage therapy (Shepherd *et al*, 2005).

Nevertheless, subgroups of patients, such as women, nonsmokers, patients with adenocarcinomas, and East Asian patients, tend to have higher objective responses and sometimes exhibit dramatic tumour shrinkage in response to these agents. It would therefore appear very important to discover biomarkers that predict gefitinib sensitivity, although EGFR protein expression, the most plausible candidate marker, does not correlate with gefitinib efficacy (Cappuzzo *et al*, 2003).

Recently, activating mutations in the TK domain of *EGFR* were reported to be strongly associated with clinical responsiveness to EGFR-TKIs (Lynch *et al*, 2004; Paez *et al*, 2004; Pao *et al*, 2004). Although these results are thought-provoking, no prospective trials have been reported to date regarding gefitinib monotherapy for advanced NSCLC with *EGFR* mutations. Here, we conducted a phase II trial to evaluate the efficacy and safety of gefitinib as first-line therapy for advanced NSCLC with *EGFR* mutations.

## MATERIALS AND METHODS

### Study design

This single-arm, phase II clinical trial recruited patients at nine institutes in north-eastern Japan. This trial consisted of two stages. First, *EGFR* mutations in exons 18–21 of the accrued patients were analysed as described below. In the second stage, only those who had *EGFR* mutations were enrolled and treated with gefitinib. The primary end point was the objective RR to an intervention of gefitinib administration (250 mg daily). Secondary end points were toxicity and survival.

### Patient eligibility

Eligible patients had histologically or cytologically confirmed stage IIIB or IV, chemotherapy-naïve NSCLCs with *EGFR* mutations. Recurrences after surgical resection were also eligible. Other eligibility criteria included: (a) age 20 years or older; (b) Eastern Cooperative Oncology Group (ECOG) performance status (PS) of 0–2; (c) measurable lesions; (d) adequate organ function (i.e., leucocyte count ⩾4000 mm^−3^, haemoglobin ⩾9.5 g dl^−1^, platelets ⩾100 000 mm^−3^, total bilirubin ⩽1.5 mg dl ^−1^, AST and ALT ⩽2 times the upper limit of the reference range, serum creatinine ⩽1.5 mg/dl ^−1^, PaO2 ⩾70 torr); and (e) life expectancy of 12 weeks or longer). Exclusion criteria comprised: (a) unstable angina, acute myocardial infarction or heart failure within the previous 3 months; (b) uncontrolled diabetes mellitus or hypertension; (c) active infection; (d) interstitial pneumonia or pulmonary fibrosis as determined from chest computed tomography (CT); (e) uncontrolled pleural effusion; (f) active gastrointestinal ulcer; (g) active metachronous cancer; (h) past history of severe hypersensitivity; (i) severe superior vena cava syndrome; and (j) pregnancy or breast-feeding. All patients were required to provide written informed consent. Trial protocol approval was obtained from the ethics committee or institutional review board (IRB) at each trial institute.

### Treatment plan

Patients received 250 mg of gefitinib orally per day. In the event of unacceptable toxicity (defined as grade 3 or more) or deterioration of PS to 3 or 4, gefitinib was ceased until this toxicity resolved and/or PS improved to ⩽grade 2 within 3 weeks. If this did not occur, treatment was terminated. In the event of grade 2 or higher interstitial lung disease (ILD), treatment was also terminated. Dose reduction was not performed. Treatment was continued unless any of the following occurred: progressive disease (PD), unacceptable toxicity (as mentioned above), the study physician decided to terminate therapy or the patient withdrew consent. No systematic anticancer treatment, radiotherapy or pleurodesis was permitted during the trial. Salvage regimens were not restricted for patients with PD or those leaving the protocol.

### Evaluation of efficacy and toxicity

Pretreatment evaluations consisted of the following: complete medical history, determination of PS, physical examination, haematologic and biochemical profiles, arterial blood gas examination, ECG, spirometry, chest X-ray, bone scan, CT of the chest, ultrasound or CT of the abdomen, and magnetic resonance imaging or CT of the whole brain. Evaluations performed during treatment included a weekly chest X-ray, biochemistry, complete blood count (including platelet and differential leucocyte counts), physical examination, determination of PS, and toxicity assessment. Moreover, for the early detection of ILD, spirometry and helical CT scan of the chest were performed once every 2 weeks for the initial 4 weeks. Imaging studies were scheduled every month to assess objective response.

Response evaluation criteria in solid tumours (RECIST) guidelines were used for evaluation of antitumour activity (Therasse *et al*, 2000). Complete response (CR) was defined as the complete disappearance of all clinically detectable tumours for at least 4 weeks. Partial response (PR) was defined as a ⩾30% decrease in the sum of the longest diameters of the target lesions for a minimum of 4 weeks with no new area of malignant disease. Progressive disease indicated at least a 20% increase in the sum of the longest diameter of the target lesions or a new malignant lesion. Stable disease (SD) was defined as insufficient shrinkage to qualify for PR and insufficient increase to qualify for PD. The minimum interval to qualify for SD was defined as 8 weeks. Responses were evaluated by the physician in charge and confirmed by independent reviewers at an extramural conference. Toxicity was graded according to the National Cancer Institute Common Toxicity Criteria (NCI-CTC) version 2.0. (National Cancer Institute, 1999).

### Mutational analysis of the *epidermal growth factor receptor* gene

Tumour specimens were obtained during diagnostic or surgical procedures. For patients with recurrences after surgical resection, mutation status was analysed in specimens of the original primary sites. Biopsied or surgically resected specimens were fixed with formalin and embedded in paraffin. Whole paraffin-embedded tissue blocks or ⩾4 slices (5-*μ*m thick) from blocks which were confirmed by each institute's pathologist to contain adequate malignant tumour were sent to First Department of Medicine, Hokkaido University. Genomic DNA was isolated from specimens using a DNeasy Tissue kit (Qiagen, Valencia, CA, USA) according to the manufacturer's instructions. For mutational analysis of the kinase domain of the *EGFR*, exons 18–21 were amplified with four pairs of primers as described previously (Paez *et al*, 2004), using a HotStarTaq DNA polymerase kit (Qiagen). Polymerase chain reaction products were purified with a PCR purification kit (Qiagen) and sequenced directly with an Applied Biosystems BigDye Terminator kit v3.1 (Applied Biosystems, Foster City, CA, USA) with an ABI PRISM 310 Genetic Analyzer. Both the forward and reverse sequences were analysed by BLAST, and chromatograms were manually reviewed. If the obtained sequences included mutation sequences, PCR amplification and sequencing analysis were repeated to confirm the results. Only the following mutations described in previous reports (Lynch *et al*, 2004; Paez *et al*, 2004; Pao *et al*, 2004) were regarded as mutation positive in the present trial; G719X in exon18, deletions in or near E746-A750 in exon 19, and L858R and L861Q in exon 21.

### Statistical analysis

Simon's two-stage minimax design was used to determine the sample size and decision criteria for this phase II trial (Simon, 1989). With a target activity level of 70% (P_1_) and minimum RR of interest set at 30% (P_0_), we needed 14 evaluable patients to accept the hypothesis and a 5% significance level to reject it with 90% power. Assuming an inevaluability rate of ⩽15%, we projected an accrual of 16 patients. Progression-free survival (PFS) was defined as the interval between enrolment in this trial and the date of documented disease progression or death from any cause. Overall survival (OS) was defined as the interval between enrolment in this trial and death from any cause. If a patient was lost to follow-up, that patient was censored at the last date of contact. Median overall and progression-PFS were estimated by the Kaplan–Meier analysis method (Kaplan and Meier, 1958). All patients who were enrolled and treated with gefitinib were included in both efficacy and toxicity analyses. Data were updated as of 15 June 2006.

## RESULTS

### Characteristics of patients undergoing *epidermal growth factor receptor* mutation analysis

From November 2004 to January 2006, 82 patients underwent analysis of *EGFR* mutation status. Patient characteristics are listed in Table 1. Forty-nine patients (60%) were female. Median age was 65 years (range, 36–83 years). The most common tumour histological type was adenocarcinoma in 72 patients (87%). Thirty-eight patients (46%) were never-smokers. Tissue samples from 44 patients (54%) were obtained by transbronchial biopsy.

### Comparison of *epidermal growth factor receptor* mutation status and clinicopathological characteristics

Twenty patients (24%) had *EGFR* mutations (deletions in or near E746-A750, *n*=16; L858R, *n*=4). Epidermal growth factor receptor mutation status and patient clinicopathological characteristics are compared in Table 2. Although no significant differences were apparent between mutation status and age, sex, histology or procedure used to obtain tumour specimens, *EGFR* mutations were more frequently observed in never-smokers than in smokers (39 *vs* 11%, *P*<0.01).

### Response to gefitinib in patients with *epidermal growth factor receptor* mutations

**Figure 1:**
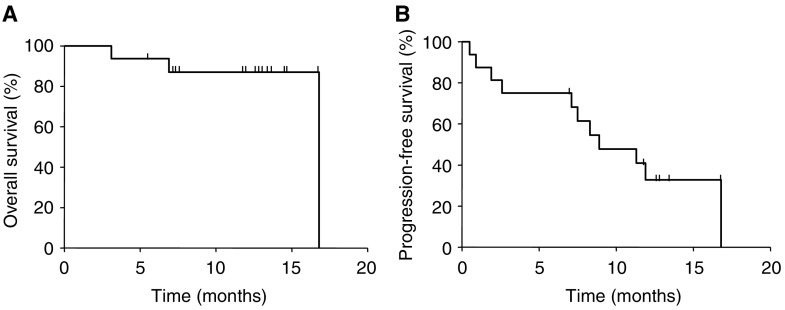
(**A**) Overall survival and (**B**) PFS of all eligible patients (*n*=16) were calculated according to the Kaplan–Meier method. Median survival time has not yet been reached and median PFS was 8.9 months (95% CI, 6.7–11.1 months).

Of 20 patients who had *EGFR* mutations, four were not enrolled to the subsequent phase II trial. One patient withdrew his consent before enrolment, one had no appropriate measurable lesions, and two were misclassified when staging. We therefore excluded these four patients before enrolment and assembled no more data from them. Excluding these four patients, 16 patients (median age, 68; male/female, 3/13; adenocarcinoma/squamous cell carcinoma, 15/1; current/former/never smokers, 2/1/13) were enrolled in the phase II trial and treated with gefitinib. Details of *EGFR* mutations and clinicopathologic features in patients receiving gefitinib are described in Table 3. Of the 16 enrolled patients, 12 achieved objective responses (two CR and 10 PR) with an overall RR of 75% (95% CI, 48–93%), one (6%) had SD and three (19%) had PD as the best response. Disease control rate (CR+PR+SD) was 81% (95% CI, 54–96%). Although the number of L858R patients was small (*n*=3), no significant difference was evident between type of mutation and RR (exon 19 deletions, 83 *vs* L858R, 67%; *P*=0.87). To date, only two patients have died, all owing to disease progression. Of the remaining 14 patients who are still alive, six maintain PR. Hence, MST has not been reached (Figure 1), and we instead evaluated median PFS. At the time of this analysis, with a median follow-up time of 12.7 months, median PFS was 8.9 months (95% CI, 6.7–11.1 months).

### Safety and toxicity

Toxicity was evaluated in all eligible patients (Table 4). The most common manifestations of toxicity were dermatological. One patient experienced grade 3 rash and terminated gefitinib treatment on day 81; however, she achieved CR on day 29 and no recurrence was detected until day 228 without any second-line treatment for 147 days. Another frequently experienced adverse effect was hepatotoxicity (elevated AST/ALT). One patient required a long treatment interruption because of grade 3 hepatotoxicity and she discontinued the protocol on day 266. A further patient experienced grade 3 gastrointestinal ulcer and grade 4 anaemia and terminated gefitinib treatment on day 56 because of PD.

**Figure 2:**
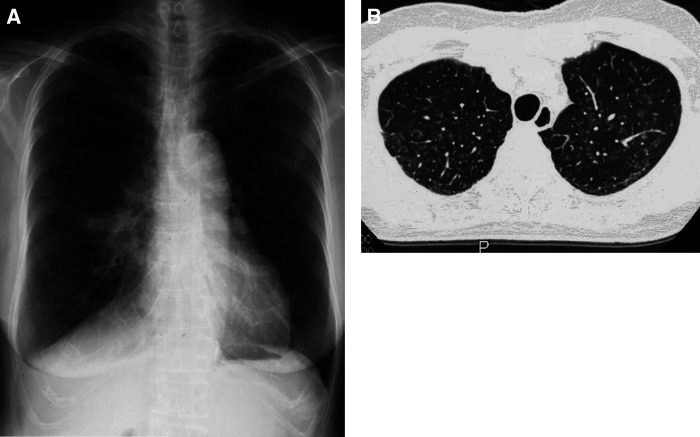
Chest X-ray (**A**) and CT (**B**) on day 30 in the patient who developed ILD. Interstitial lung disease was hardly recognisable on chest X-ray, whereas chest CT revealed a patchy, ground-glass opacity with centrilobular distribution throughout the whole lung.

Interstitial lung disease is the most problematic toxicity in gefitinib treatment in Japan (Inoue *et al*, 2003). In the present trial, one patient experienced grade 1 ILD on day 30, leading to the termination of gefitinib treatment. This patient was asymptomatic; ILD was barely detectable on chest X-ray and was recognised only on chest CT, which revealed a patchy, ground-glass opacity with centrilobular distribution throughout the lung (Figure 2). This was assumed to be hypersensitivity-type ILD, and it improved without steroid administration.

### Second-line treatment after disease progression

Of 10 patients who became refractory to gefitinib owing to disease progression, six received second-line treatment. Two received carboplatin plus paclitaxel; one had PR and another had PD as second-line treatment. Another two patients received gemcitabine plus vinorelbine; one patient had PR and another had PD as second-line treatment. A further patient terminated gefitinib monotherapy because of grade 3 hepatotoxicity, recovered after 8 weeks treatment interruption and then resumed gefitinib. The other patient, in whom PD was diagnosed owing to development of a new bone metastasis, resumed gefitinib monotherapy after radiation therapy to the bone metastasis, as his primary and other lesions were still controlled by gefitinib. The latter two patients also remained alive at the most recent follow-up.

## DISCUSSION

As two separate groups reported somatic mutations of the *EGFR* TK domain in NSCLC in May 2004 (Lynch *et al*, 2004; Paez *et al*, 2004), over 15 studies on mutational analysis of this domain (exons 18–21) in over 3000 patients have been reported by different groups around the world (Chan *et al*, 2006). The collective data indicate an overall mutation rate of 17%, but this was higher in East Asians, never-smokers, women, and patients with adenocarcinoma. Notably, patients with mutated *EGFR* had a much higher RR to gefitinib than those with wild-type *EGFR* (77 *vs* 23%). Several groups also reported prolonged time to progression (range, 7.6–21.7 months) and OS time (range, 13.0–30.5 months) in patients with *EGFR* mutations (Chou *et al*, 2005; Cortes-Funes *et al*, 2005; Han *et al*, 2005; Kim *et al*, 2005; Mitsudomi *et al*, 2005; Takano *et al*, 2005; Taron *et al*, 2005; Zhang *et al*, 2005). However, retrospective analysis of the mutation status of patients enrolled in the previous large clinical trials of *EGFR*-TKI (Bell *et al*, 2005; Eberhard *et al*, 2005; Tsao *et al*, 2005) has not demonstrated a significant relationship between presence of *EFGR* mutation and response to such agents. Hence, to elucidate the efficacy of gefitinib in patients with *EGFR* mutations, the present prospective trial is warranted.

In this phase II trial, we demonstrated an extremely high objective RR of 75% (95% CI, 48–93%), median PFS of 8.9 months, and unattained OS, consistent with previous retrospective analyses. These results were much better than those for standard platinum-containing regimens as first-line therapy (Schiller *et al*, 2002). Therefore, although the real survival benefit needs to be examined in future randomised phase III trials, our results clearly demonstrated that gefitinib has considerable activity in patients with *EGFR* mutation, even as first-line therapy.

Despite such a good response to gefitinib, retrospective analysis of INTACT trials shows that patients with *EGFR* mutation also tend to be more sensitive to platinum-based chemotherapy than those with wild-type *EGFR* (Bell *et al*, 2005). This matter also needs to be addressed in future randomised phase III trials.

To date, two phase II clinical trials of gefitinib monotherapy as first-line therapy have been performed that did not consider *EGFR* mutation status. One evaluated gefitinib treatment without any patient selection (Niho *et al*, 2006) and the other was conducted with never-smokers (Lee *et al*, 2005). Although the former showed an unimpressive RR of 30%, the latter demonstrated high gefitinib efficacy; objective response, median PFS, and 1-year survival were 69%, 33 weeks and 73%, respectively. Although their observed responses were favourable and close to those observed in the present trial, patient selection based on smoking history might have the disadvantage of excluding the smokers with *EGFR* mutations who could have the same sensitivity to gefitinib as never-smokers.

Toxicity observed in the present trial was mostly favourable when compared to previous clinical trials of gefitinib (Fukuoka *et al*, 2003; Kris *et al*, 2003; Giaccone *et al*, 2004; Herbst *et al*, 2004; Thatcher *et al*, 2005) and of standard chemotherapeutic regimens (Schiller *et al*, 2002). Interstitial lung disease is the most problematic toxicity in gefitinib treatment in Japan where an incidence of 3.5% and a fatality rate of 1.6% have been reported (Inoue *et al*, 2003; Ando *et al*, 2006). Accordingly, in the present trial, we planned to conduct biweekly chest CT for early detection of ILD during the initial treatment period. Despite one case of ILD that occurred in the present trial, the initial biweekly CT detected asymptomatic grade 1 ILD better than has been reported previously. As ILD was detected at an early stage, it may have responded to discontinuation of gefitinib.

One problem related to patient selection based on mutation status is the method of detecting *EGFR* mutations. In the present trial, we used a direct sequence method with paraffin-embedded tissues. This method is conventional but is complex, expensive, and time consuming for daily clinical practice. Recently, simple, sensitive, and rapid detection methods such as PNA-LNA clump and LightCycler PCR assay have been developed (Nagai *et al*, 2005; Sasaki *et al*, 2005) and could resolve such problems. Clinical trials using these methods are being conducted by multiple groups.

In conclusion, gefitinib treatment as first-line therapy for advanced NSCLC with *EGFR* mutations demonstrated promising activity and a good toxicity profile. Randomised phase III trials comparing gefitinib and standard platinum-based chemotherapy for patients with *EGFR* mutations are now being conducted and have the potential to change our daily clinical practice with respect to advanced NSCLC.

## Figures and Tables

**Table 1:** Characteristics of all patients whose tumours were analysed for *EGFR* mutations

**Characteristics**	**No. of patients (%)**
Total no. of patients	82
*Age* (*years*)	
Median	65
Range	36–83
	
*Sex*	
Male	33 (40)
Female	49 (60)
	
*Histology*	
Adenocarcinoma	72 (87)
Squamous cell carcinoma	4 (5)
Large cell carcinoma	3 (4)
Other	3 (4)
	
*Smoking history*	
Current	28 (34)
Former	16 (20)
Never	38 (46)
	
*Tissue obtained by*	
Surgery	30 (36)
Transbronchial biopsy	44 (54)
Other biopsies	8 (10)

Abbreviation: EGFR=epidermal growth factor receptor.

**Table 2:** Relationship between *EGFR* mutation status and clinicopathological characteristics

	**Mutated**	**Wild type**	
**Characteristics**	**No. of patients (%)**	**No. of patients (%)**	******P******
Total no. of patients	20 (24)	62 (76)	
Age (range)	67 (36–82)	62 (47-83)	0.10^a^
			
*Sex*			
Male	5 (15)	28 (85)	0.09^b^
Female	15 (31)	34 (69)	
			
*Histology*			
Adenocarcinoma	19 (26)	53 (74)	0.24^b^
Non-adenocarcinoma	1 (10)	9 (90)	
			
*Smoking history*			
Smoker	5 (11)	39 (89)	0.003^b^
Never-smoker	15 (39)	23 (61)	
			
*Specimen*			
Surgery	7 (23)	23 (77)	0.54^b^
Biopsy	13 (25)	39 (75)	

Abbreviation: EGFR=epidermal growth factor receptor.

a Student’s *t*-test.

b Fisher’s exact test.

**Table 3:** Patients with *EGFR* mutation who were enrolled in the phase II trial

**No.**	**Age**	**Sex**	**Smoking history**	**Histology**	**Stage**	**Specimen**	***EGFR* mutation site**	**Nucleotide change**	**Amino-acid change**	**Response**	**TTP (month)**	**OS (month)**
1	47	F	Never	Ad	IV	Surgery	Exon 19	2235–2249 del	del E746-A750	CR	16.8+^a^	16.8+
2	72	F	Never	Ad	Recurrence	TBB	Exon 19	2237–2254 del	del E746-S752insV	CR	8.3	14.8+
3	68	F	Never	Ad	IV	LB	Exon 19	2236–2250 del	del E746-A750	PR	7.5	14.7+
4	63	F	Former	Sq	IV	TBB	Exon 19	2236–2250 del	del E746-A750	PD	0.5	6.9
5	80	F	Never	Ad	IV	TBB	Exon 19	2236–2250 del	del E746-A750	SD	8.9	13.6+
6	78	M	Current	Ad	Recurrence	Surgery	Exon 21	2573 T>G	L858R	PR	13.4+	13.4+
7	67	F	Never	Ad	IIIb	TBB	Exon 19	2236–2250 del	del E746-A750	PR	12.8+	12.8+
8	65	F	Never	Ad	IV	TBB	Exon 19	2240–2257 del	del L747-P753insS	PR	11.9	11.9+
9	51	F	Never	Ad	IV	TBB	Exon 19	2240–2257 del	del L747-P753insS	PR	11.3	12.7+
10	57	F	Never	Ad	IV	TBB	Exon 19	2240–2257 del	del L747-P753insS	PR	12.7+	12.7+
11	83	F	Never	Ad	Recurrence	Surgery	Exon 21	2573 T>G	L858R	PR	11.7+	11.7+
12	81	F	Never	Ad	IIIb	TBB	Exon 19	2240–2257 del	del L747-P753insS	PR	2.6	7.5+
13	52	F	Never	Ad	IV	TBB	Exon 19	2235–2249 del	del E746-A750	PR	7.1	7.1+
14	70	M	Never	Ad	IV	Surgery	Exon 21	2573 T>G	L858R	PD	1.9	3.1
15	69	F	Never	Ad	Recurrence	Surgery	Exon 19	2236–2250 del	del E746-A750	PR	7.0+	7.0+
16	65	M	Current	Ad	IV	PLB	Exon 19	2236–2250 del	del E746-A750	PD	0.9	5.4+

Abbreviations: TTP=time to progression; OS=overall survival; F=female; M=male; Ad=adenocarcinoma; del=deletion; Sq=squamous cell carcinoma; TBB=transbronchial biopsy; ins=insertion; CR=complete remission; LB=liver biopsy; PR=partial remission; SD=stable disease; PD=progressive disease; PLB=percutaneous lung biopsy; EGFR=epidermal growth factor receptor.

a Still alive with no progression at the time of data collection.

**Table 4:** Major toxicities associated with gefitinib treatment

	**No. patients (%)**			
**Adverse event**	**Grade 1**	**Grade 2**	**Grade 3**	**Grade 4**
*Haematologic toxicity*				
Leucopenia	0 (0)	1 (6)	0 (0)	0 (0)
Neutropenia	1 (6)	0 (0)	0 (0)	0 (0)
Anaemia	2 (13)	0 (0)	0 (0)	1 (6)
Thrombocytopenia	0 (0)	0 (0)	0 (0)	0 (0)
				
*Nonhaematologic toxicity*				
Rash	2 (13)	5 (31)	1 (6)	0 (0)
Dry skin	4 (25)	0 (0)	0 (0)	0 (0)
Pruritus	6 (38)	0 (0)	0 (0)	0 (0)
Nail changes	0 (0)	1 (6)	0 (0)	0 (0)
Stomatitis	2 (13)	1 (6)	0 (0)	0 (0)
Gastric ulcer	0 (0)	0 (0)	1 (6)	0 (0)
Anorexia	4 (25)	1 (6)	1 (6)	0 (0)
Nausea	1 (6)	1 (6)	1 (6)	0 (0)
Vomiting	0 (0)	1 (6)	0 (0)	0 (0)
Diarrhoea	6 (38)	1 (6)	0 (0)	0 (0)
Constipation	2 (13)	0 (0)	0 (0)	0 (0)
Elevated bilirubin	2 (13)	1 (6)	0 (0)	0 (0)
Elevated AST/ALT	3 (19)	2 (13)	2 (13)	0 (0)
Elevated ALP	4 (25)	1 (6)	0 (0)	0 (0)
Elevated creatinine	2 (13)	0 (0)	0 (0)	0 (0)
ILD	1 (6)	0 (0)	0 (0)	0 (0)

Abbreviations: ILD=interstitial lung disease; ALP=alkaline phosphatase; AST/ALT=aspartate aminotransferase/alanine aminotransferase.
